# Multi-Signal Detection Framework: A Deep Learning Based Carrier Frequency and Bandwidth Estimation

**DOI:** 10.3390/s22103909

**Published:** 2022-05-21

**Authors:** Meiyan Lin, Xiaoxu Zhang, Ye Tian, Yonghui Huang

**Affiliations:** 1 National Space Science Center, Chinese Academy of Sciences, Beijing 100190, China; linmeiyan18@mails.ucas.ac.cn (M.L.); zhangxiaoxu19@mails.ucas.ac.cn (X.Z.); tianye171@mails.ucas.ac.cn (Y.T.); 2 University of Chinese Academy of Sciences, Beijing 100190, China

**Keywords:** multi-signal detection, deep learning, cognitive radio, parameter estimation, non-cooperative communication

## Abstract

Multi-signal detection is of great significance in civil and military fields, such as cognitive radio (CR), spectrum monitoring, and signal reconnaissance, which refers to jointly detecting the presence of multiple signals in the observed frequency band, as well as estimating their carrier frequencies and bandwidths. In this work, a deep learning-based framework named SigdetNet is proposed, which takes the power spectrum as the network’s input to localize the spectral locations of the signals. In the proposed framework, Welch’s periodogram is applied to reduce the variance in the power spectral density (PSD), followed by logarithmic transformation for signal enhancement. In particular, an encoder-decoder network with the embedding pyramid pooling module is constructed, aiming to extract multi-scale features relevant to signal detection. The influence of the frequency resolution, network architecture, and loss function on the detection performance is investigated. Extensive simulations are carried out to demonstrate that the proposed multi-signal detection method can achieve better performance than the other benchmark schemes.

## 1. Introduction

With the advent of the Internet of Things (IoT), the electromagnetic spectrum scarcity has become an increasingly important problem [1,2,3]. Cognitive radio (CR) is an encouraging solution to resolve spectrum scarcity in wireless communications using dynamic spectrum access (DSA) [4,5]. In CR, two common spectrum sharing strategies exists: (i) the secondary users (SUs) can utilize spectrum that is not used by the primary users (PUs); (ii) the SUs are allowed to transmit when the PUs are transmitting, by superimposing its transmission to the primary user (namely, superposition coding) [6,7,8]. In the former spectrum sharing paradigm, it is needed to sense the spectrum to obtain the usage status of frequency resources. Building smart spectrum sensing products in the license-free band to monitor and analyze the electromagnetic spectrum would be of great commercial value, especially in the IoT era where wireless device density increases significantly. Furthermore, in civilian and military applications such as spectrum monitoring and management [9], as well as battlefield electromagnetic spectrum situational awareness [10,11], signal detection and relevant parameters estimation are indispensable. It will benefit mastery of spectrum usage in the observation frequency band.

Multi-signal detection is aiming to jointly determine the existence of signals in a specific wideband, and estimate signal parameters such as the number of separable signals, center frequencies, and bandwidths. This is different from most spectrum sensing works, which only estimate signal “presence” or “absence”.

### 1.1. Related Works and Motivations

Many signal detection algorithms have been studied in the past decades, including energy detection (ED) [12,13], matched filtering detection [14,15], cyclostationary feature detection [16,17] and eigenvalue based detection [18]. Although the matched filtering and cyclostationary feature detections exhibit good performance, these schemes require prior information about the transmitted signal such as the transmitted period, which is not always available in practice. In contrast, energy detection is a simple and effective method to detect the presence of signal and requires no prior knowledge of the transmitted signal, but is susceptible to noise power [19]. Nevertheless, most of the above work only focuses on detecting the presence of signal (binary detection decision).

For jointly detecting the presence of signal, as well as estimating the bandwidth and center frequency, several algorithms that are based on a threshold have been proposed [20,21,22]. Threshold setting is a key issue because the threshold directly affects the performance of the detection algorithms. Therefore, many methods have been proposed for determining the detection threshold, such as measuring noise power [23], analyzing noise histogram, or spectrum histogram [24]. However, these methods possess some drawbacks. For instance, they do not perform well when the noise power varies across the spectrum. Moreover, some of the methods require a priori knowledge of the noise statistics for the threshold estimation. A localization algorithm based on double-thresholding (LAD) is proposed for detecting and localizing multiple signals in the frequency domain [25,26]. The LAD method uses two thresholds, upper and lower. The lower threshold is used to avoid signal separation and the upper threshold helps to avoid false detections. However, the LAD method has a trade-off between the performance of the detection probability and the false alarm probability, especially in the case of a low signal-to-noise ratio (SNR).

With the advent of the age of artificial intelligence, deep learning and neural network (NN) have been rapidly improved and have numerous applications. For signal detection, several methods utilizing neural networks have been put forward [27,28,29]. In [27], a convolutional neural network (CNN) is proposed to learn the energy-correlation features from the signal sample covariance matrix. A deep learning framework, namely DeepMorse, is proposed to detect morse signals in wideband spectrum data without prior knowledge [28]. In [29], a deep learning-based detector is proposed, which consists of CNN, a self-attention (SA) module, and a gate recurrent unit (GRU). Compared with traditional detection algorithms, the deep learning-based algorithms exhibit superior performance due to the NN’s powerful ability to learn key features from the signal samples. Unfortunately, these methods only detect the presence of the signal and cannot estimate the relevant parameters. Furthermore, a Q-learning-based method is presented in [30] to identify those temporarily unused frequency ranges. In [30], the epsilon-greedy action selection method is also adopted to indicate the next monitoring channel. In [31], the object detection network named single shot multibox detector (SSD) is developed for detecting signals by using the time-frequency spectrogram. Similarly, the work in [32] has employed a downscaled Faster region-based convolutional neural network (Faster-RCNN) to detect and localize Wi-Fi signals when uninteresting signals cause RF interference (RFI). However, these methods are difficult to accurately obtain the time and frequency information of the signal using the bounding box of object detection.

Different from the object detection task, the goal of scene parsing is to classify images at the pixel level, and obtain the category of each pixel. Most scene parsing frameworks are based on a full convolutional network (FCN) [33]. The works of [34,35] have improved the performance of the original FCN, and now these networks have been successfully applied to complex scene parsing tasks. In [35], a pyramid scene parsing network (PSPNet) is proposed for the scene parsing task, which utilizes the global context information of different regions through pyramid pooling. In [36], the FCN has been applied to the detection task of real satellite signals. Nevertheless, the FCN classifies the pixels in the input sequence independently, lacking the relationship between pixels, and may lose the detailed information of features. Motivated by the task of scene parsing, the multi-signal detection task is highly analogous to image segmentation, detecting whether each frequency bin in the broadband power spectrum contains a signal.

### 1.2. Contributions and Organization

To develop a multi-signal detector, several challenges should be addressed. First, the signals in electromagnetic space are increasing and changing dynamically, ranging from several to dozens; second, various uncorrelated signals are usually transmitted simultaneously in different modulation types, and may even contain burst signals; third, the background noise in the electromagnetic environment increases significantly, and the dynamic range of the signal is large, which makes it difficult to detect the weak signal.

In this work, a multi-signal detection framework based on deep learning named SigdetNet is proposed, which can perform two major tasks simultaneously: (i) detect multi-signal in the frequency band of interest; (ii) estimate their center frequencies and bandwidths. By taking the power spectrum as the network’s input, the proposed framework transform the multi-signal detection problem into a scene parsing problem, performing pixel-wise classification. The Welch’s periodogram method [37] is used to obtain the power spectrum, which can reduce the variance in the power spectral density (PSD). Moreover, logarithmic transformation is applied to the PSD, scaling the numerical range of the PSD magnitudes, thereby enhancing the weak signals. In particular, a convolutional encoder-decoder network embedded with the pyramid pooling module (PPM) is constructed to extract informative features related to the signal detection task. The convolutional encoder-decoder network has been proved to have the ability to extract high-level representative features from noisy [29]. While, the PPM can capture multi-scale information by fusing different pyramid level features without significantly increasing the complexity. The main contributions of this paper are summarized as follows:

1. We develop a relatively complete deep learning-based framework for multi-signal detection, including signal pre-processing, signal enhancement, feature extraction using NN, and post-processing.
2. Extensive simulations are carried out to demonstrate the superiority of our proposed method compared with the benchmark detectors. In addition, the influence of design parameters, e.g., frequency resolution, network architecture, and loss function, on the performance of the proposed method are investigated.

This paper is organized as follows. Section 2 introduces the mathematical model on multi-signal detection. In Section 3, the proposed method is introduced in detail. In Section 4, evaluation criteria, datasets, and experiments are given. Section 5 reveals the results of the experiments. At last, Section 6 summarizes the whole paper.

Notations: In this paper, superscripts ${\left(\cdot \right)}^{T}$ denote the transpose operation. ℜ(·) denote the real part of a complex number. Boldface lowercase letters such as a, b denote vectors, and boldface uppercase letters such as A, B denote matrices.

## 2. Problem Statement

In this paper, we consider a non-cooperative communication scenario, in which multiple heterogeneous transmitters are emitting wireless signals at different center frequencies with different modulation types, such as amplitude shift keying (ASK) modulation, phase shift keying (PSK) modulation, frequency shift keying (FSK) modulation, Gaussian minimum shift keying (GMSK) modulation, and so on. The *i*-th single transmission signal $s_{i}\left(t\right)$ can be generally presented as

(1) $$s_{i}\left(t\right)=\sqrt{2}ℜ\left\{\sum_{m}a_{m}g\left(t-mT_{i}\right)e^{j(2\pi f_{i}t+\phi_{i})}\right\}$$

where ℜ(·) denotes the real part of a complex number; $a_{m}=a_{mi}+ja_{mq}$ is the complex symbol sequence; g(t) is the pulse shape function. The bandwidth, carrier frequency, initial phase, and symbol period of the *i*-th signal are denoted by $B_{i}$, $f_{i}$, $\phi_{i}$, and $T_{i}$ respectively.

Assuming that a wideband receiver captures the radio frequency (RF) data at a sampling rate $F_{s}$ and duration *T*. Multiple different wireless communication signals $s_{i}\left(t\right)$ are captured together by the receiver. While modulated signals overlap in the time domain, they would exhibit various shapes and distributions in the frequency domain. The discrete-time series r(n) obtained by the receiver is composed of $N_{sig}$ signals, which is defined as

(2) $$r\left(n\right)=\sum_{i=1}^{N_{sig}}s_{i}\left(n\right)+w\left(n\right)$$

where $N_{sig}$ is the number of signals; $s_{i}\left(n\right)$ is the discrete form of the signal $s_{i}\left(t\right)$; w(n) is the receiver noise, which is modeled as Additive White Gaussian Noise (AWGN). The parameters such as the power, modulation type, carrier frequency, and bandwidth of each signal $s_{i}\left(n\right)$ are different and are unknown to the receiver. Moreover, in the electromagnetic environment, multiple irrelevant signals are usually transmitted simultaneously in different frequency bands and do not overlap in the frequency domain. In this work, our goal is to develop a deep learning-based method for RF spectrum analysis, focusing on the presence detection of signals within the observed band, as well as estimating their frequencies and bandwidths. This is a wideband signal detection problem because the sampling bandwidth of the receiver is much wider than that of any individual signal bandwidth (such that multi-signal may appear within the sampling bandwidth).

**Figure 1:**
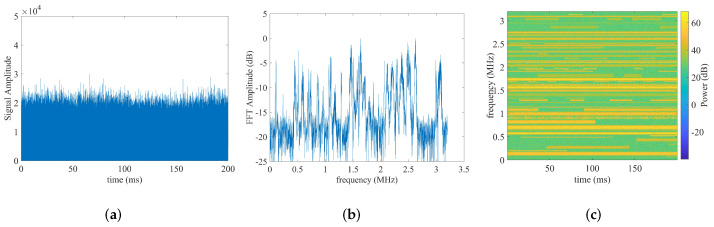
The time content, frequency content, and spectrogram of an example RF capture with a sampling rate of 6.4 MHz and a duration of 200 ms, respectively. (**a**) signal amplitude vs. time; (**b**) FFT magnitude vs. frequency; (**c**) time-frequency spectrogram.

For illustration, Figure 1 shows the time and frequency content of an example wideband capture with $F_{s}=6.4$ MHz and T=200 ms. The signal amplitude is plotted as a function of time in Figure 1a, the fast Fourier transform (FFT) amplitude is plotted as a function of frequency in Figure 1b, and the time-frequency representation of the spectrum is plotted as a function of both time and frequency in Figure 1c. The example captured RF data in Figure 1 contains 52 narrowband signals, including burst signals.

## 3. Proposed Detection Framework

**Figure 2:**
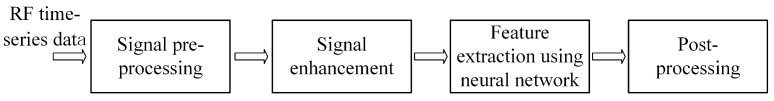
The proposed framework for multi-signal detection.

In this work, a deep learning-based framework is proposed to detect the presence of signals in the observation frequency band, as well as estimate their carrier frequencies and bandwidths. The proposed framework, named SigdetNet, consists of four stages, which referred to signal pre-processing, signal enhancement, feature extraction using neural network, and post-processing respectively, as shown in Figure 2. The framework takes the received RF data as the input, and predicts the number, carrier frequencies, and bandwidths of signals.

### 3.1. Signal Pre-Processing

In this work, the power spectrum is used as the network’s input format to obtain the frequency-wise energy distribution. The PSD estimation techniques are generally categorized into parametric and non-parametric techniques. The parametric PSD estimators, such as the Burg’s method [38] and the Yule–Walker method [39], try to fit a parametric model to the signal by minimizing a given cost function [40,41]. In the parametric techniques, it is sensitive to the choice of model order *P* to obtain accurate power spectrum estimation. In contrast to parametric techniques, the non-parametric techniques do not make any assumptions about the data-generating process or model, e.g., the autoregressive model [42]. The common non-parametric techniques available in the literature include the periodogram [43], the modified periodogram [44], Bartlett’s method [45], and Welch’s method [37]. Among the non-parametric techniques, Welch’s method can reduce the variance in the PSD estimation and improve the estimation quality. Welch’s method eliminates the tradeoff between spectral resolution and variance, and is widely used in spectrum sensing [46,47,48,49,50,51]. In [46], Sarvanko et al. generalized the theoretical foundations of ED for the case of Welch’s periodogram, and analyze the performance of spectrum sensing in Gaussian channels, concluding that Welch’s method for PSD estimation performs better than the classical periodogram for detecting narrowband signals. Hence, Welch’s method is selected to estimate the PSD.

To obtain Welch’s power spectrum, the received signal r(n) with length *N* is divided into *L* segments of length *M*, allowing overlapping between consecutive segments. The *l*-th segment is shown in Equation (3). Note that the length of r(n) is equal to $N=T\cdot F_{s}$.

(3) $$\begin{array}{c} r_{l}\left(n\right)=r\left(n+lD\right)\;n=0,1,\cdots ,M-1;\;l=0,1,\cdots ,L-1. \end{array}$$

where the overlap between segments is M−D, 0<D≤M; lD is the starting point for the *l*-th segment.

Then, a window function, w(n), is applied to each segment. The periodogram for the *l*-th segment is

(4) $$\begin{array}{cc} p_{l}\left(k\right) & =\frac{1}{MU}{\left|\sum_{n=0}^{M-1}r_{l}\left(n\right)w\left(n\right)e^{-j2\pi (kn/K_{fft})}\right|}^{2}\;k=0,1,\cdots ,K_{fft}-1 \end{array}$$

where $K_{fft}$ corresponds to the number of points considered in the FFT for the periodograms; *U* is the normalization factor to ensure that the window function has a unitary power, namely:

(5) $$U=\frac{1}{M}\sum_{n=0}^{M-1}w^{2}\left(n\right)$$

The values of the individual periodogram obtained from the received signal, r(n), are contained in a matrix of size $L\times K_{fft}$, defined as

(6) $$\tilde{P}≜{\left[\left({\tilde{p}}^{1}\right)\;\left({\tilde{p}}^{2}\right)\;\cdots \;\left({\tilde{p}}^{L}\right)\right]}^{T}$$

where superscript *T* denotes the transpose operation, and vectors ${\tilde{p}}_{l}\in R^{K_{fft}\times 1}$ are defined as

(7) $${\tilde{p}}^{l}≜{\left[p_{l}\left(0\right)\;p_{l}\left(1\right)\;\cdots \;p_{l}\left(K_{fft}-1\right)\right]}^{T}$$

Finally, the Welch’s power spectrum corresponds to the average of the *L* modified periodograms

(8) $$p\left(k\right)=\frac{1}{L}\sum_{l=0}^{L-1}p_{l}\left(k\right)\;k=0,1,\cdots ,K_{fft}-1$$

The equivalent vector form of Welch’s power spectrum $p\in R^{K_{fft}\times 1}$ is defined as

(9) $$p≜{\left[p\left(0\right)\;p\left(1\right)\;\cdots \;p\left(K_{fft}-1\right)\right]}^{T}$$

### 3.2. Signal Enhancement

The resulting Welch’s power spectrum p reflects the energy distribution in the frequency domain. Signals with high SNR exhibit high values in the vector p, while signals with low SNR may be hidden beneath the background (i.e., noise). Logarithmic transformation is widely used in image enhancement, which converts a narrow range of low input grey level values into a wider range of output values to reveal more detail [52]. To distinguish the signal from the background and scale the numerical range of the spectral, the logarithmic transformation is assigned to each frequency bin of the vector p to obtain an enhanced output $p_{e}$:

(10) $$p_{e}\left(k\right)=\frac{\mathrm{lg}(c\cdot p(k))}{\mathrm{lg}(c+1)}\;k=0,1,\cdots ,K_{fft}-1$$

where the *c* is usually set to 1.

**Figure 3:**
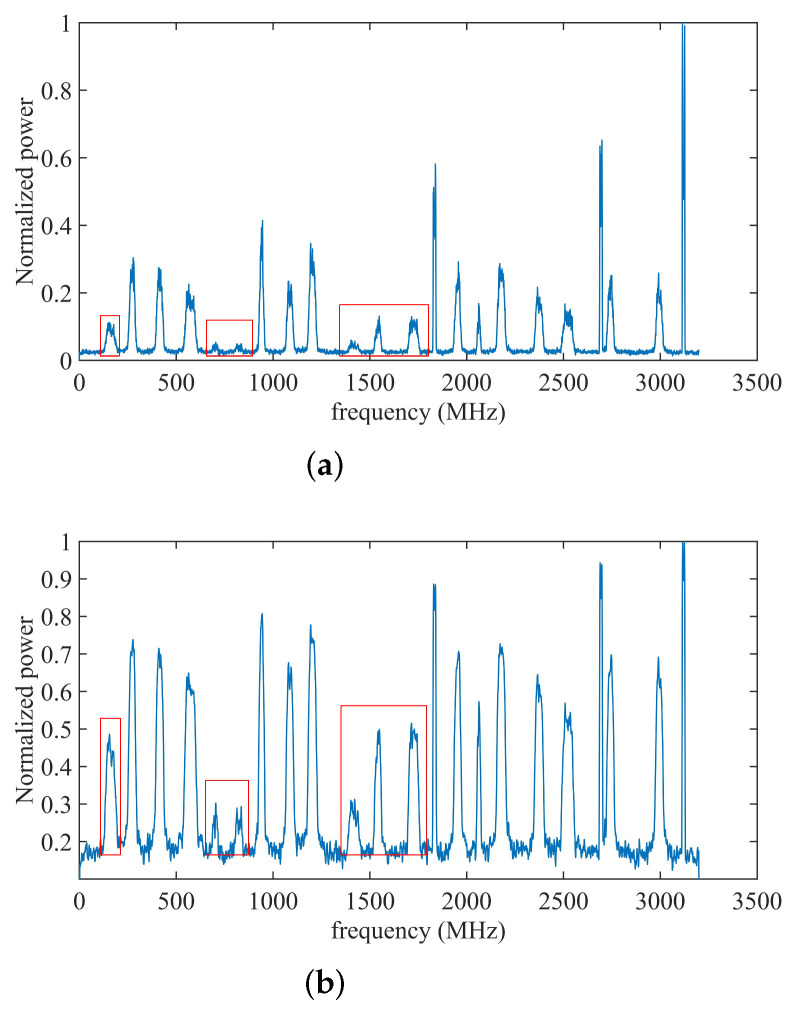
Signal enhancement using logarithmic transformation. (**a**) before enhancement; (**b**) after enhancement.

The enhanced $p_{e}$ is then normalized to [0,1] by Min-Max normalization:

(11) $$p_{e}^{\prime }=\frac{p_{e}-min\left(p_{e}\right)}{max\left(p_{e}\right)-min\left(p_{e}\right)}$$

where the vector $p_{e}^{\prime }\in R^{K_{fft}\times 1}$ is the normalized results of the $p_{e}\in R^{K_{fft}\times 1}$. Figure 3 presents the Welch’s power spectrum p (normalized) and the power spectrum after logarithmic transformation $p_{e}^{\prime }$ (normalized), respectively. It can be seen that the weaker signals are enhanced after logarithmic transformation, as shown in Figure 3 marked by the red box.

### 3.3. Feature Extraction

**Figure 4:**
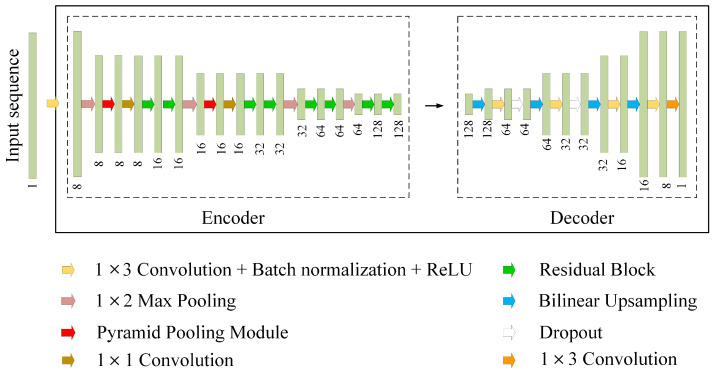
Overview of the proposed network.

To jointly detect the presence of signals, as well as estimate the carrier frequency and bandwidth of each signal in the wideband input data, a one-dimensional convolutional encoder-decoder network is developed, as illustrated in Figure 4. In addition, the pyramid pooling module (PPM) is embedded in the network to fuse multi-scale features. The proposed network takes the enhanced Welch’s power spectrum $p_{e}^{\prime }$ as input, and outputs a spectrum segmentation mask $\hat{y}$. The goal of the network is to assign a category label to each pixel in the input power spectrum, which includes two categories: signal and background. Details of the proposed network are presented below.

**Figure 5:**
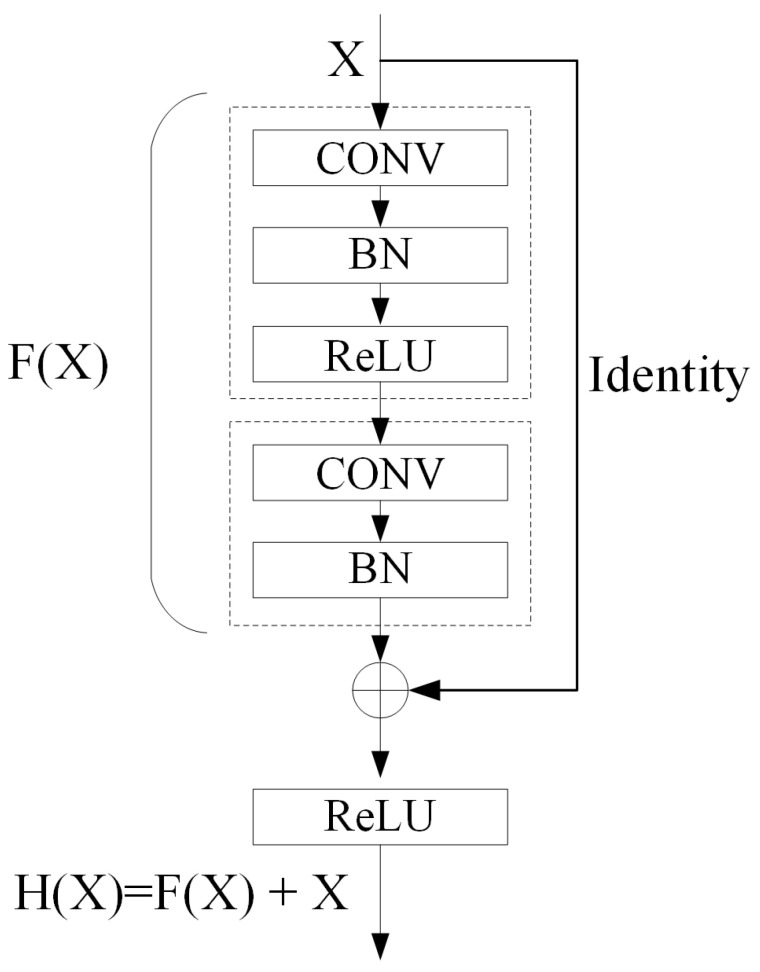
The residual block structure.

The encoder part in the proposed network performs convolution with a kernel bank to produce a set of feature maps to extract high-order information that can describe the characteristics of the input. To solve the degradation problem in deeper networks, a residual learning framework, ResNet, is proposed in [53]. The structure of the residual block in the ResNet is shown in Figure 5. Suppose the fitting function of the stacked nonlinear layers is F(X), and the target fitting function H(X) can be decomposed into F(X)+X. The designed encoder contains one convolutional layer, eight residual blocks, and two embedding pyramid pooling modules. Each convolutional layer is followed by batch normalized (BN) to facilitate training [54]. Following that, the nonlinear activation function of the rectified linear unit (ReLU) max(0,x) is applied. In addition, max-pooling with 1×2 window and stride equal to 1 is performed, and the resulting output is downsampled by a factor of 2.

**Figure 6:**
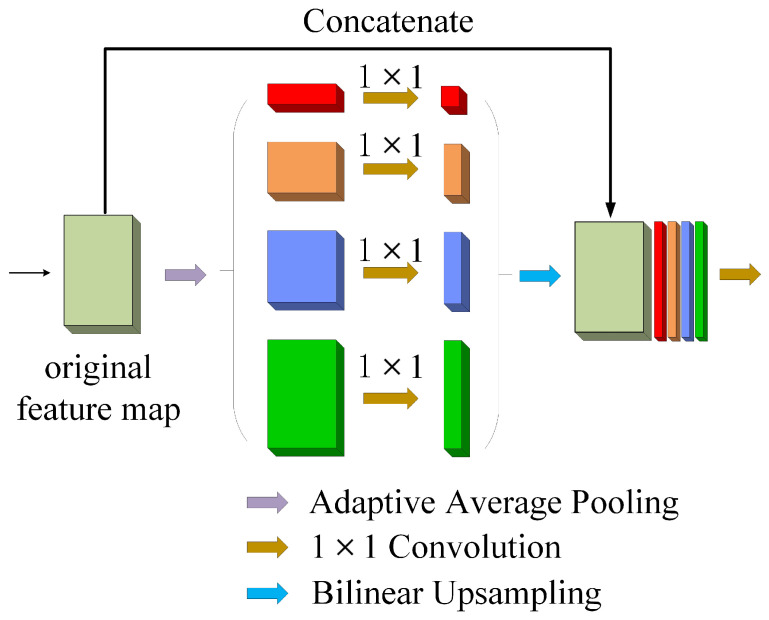
The pyramid pooling module structure.

The pyramid pooling module can fuse multi-scale features at different pyramid levels and synthesize context information. Context information can take into account the correlation between pixels instead of making independent predictions for pixels in the input sequence. The structure of the pyramid pooling module is illustrated in Figure 6. The pyramid pooling module consists of four steps, including adaptive average pooling, convolution, bilinear upsampling, and concatenation operations. Feature maps at different pyramid scales can be obtained by adaptive average pooling. Then 1×1 convolution layer is added to each pyramid level to set the number of channels to 1. The convoluted feature maps are further interpolated using bilinear upsampling to match the size of the original feature map. The original feature map is finally concatenated with the four upsampled feature maps so that multi-scale features can be used to maintain global features. In the adaptive average pooling layer, the pooling size of 1×1, 1×2, 1×3, and 1×6 are used in our settings.

The compressed high-order encoder features are blurred, and boundary detail has been lost. Therefore, a feature recovery network (decoder) is designed to map the low-resolution encoder feature maps to full-input resolution feature maps for pixel-wise prediction. The decoder upsamples the feature maps by using the bilinear upsampling. Following that, a trainable convolution layer is applied after each bilinear upsampling to recover the boundary details of the segmentation mask. In addition, a dropout operation is added to activate the part of the weights to reduce parameters and thus alleviate overfitting. The output is converted to the probability that each pixel is a signal or background using the Softmax activation function.

Instead of performing intensive pixel-level classification using cross-entropy (CE) loss, the network is trained with Dice loss [55], which is based on the Dice coefficient *D*. The Dice loss and Dice coefficient are defined by Equations (12) and (13), respectively.

(12) $$loss_{Dice}=1-D$$

(13) $$D=\frac{2\hat{y}\cdot y}{\hat{y}+y}$$

where $\hat{y}$ and y denote the network’s output and ground-truth, respectively. The Dice coefficient *D* describes the similarity between two vectors, and its value ranges from 0 to 1. The larger the value of *D*, the stronger the similarity between the two vectors. Compared with CE loss, Dice loss can solve the problem of uneven distribution of positive and negative samples. For example, if the proportion of pixels with background is larger than that of pixels with the signal. Then the unevenness of positive samples (signal) and negative samples (background) will cause the learning process to fall into the local minima of the loss function, making the network biased towards negative samples.

### 3.4. Post-Processing

Each value in the predicted spectrum segmentation mask $\hat{y}$ represents the probability that the pixel contains a signal. By setting a binarization threshold γ on the predicted mask $\hat{y}$ to obtain a binarized segmentation mask, and search the lower and upper frequency bounds of signals. In the binarized segmentation mask, each sub-sequence with consecutive “1” is a detected signal, and its lower and upper frequency bounds (equivalent to a center frequency and bandwidth estimate) can be determined. We locate the start index ${\hat{I}}_{i}^{start}$ and end index ${\hat{I}}_{i}^{end}$ of each consecutive “1” region in the binarized segmentation mask. The start index ${\hat{I}}_{i}^{start}$ and end index ${\hat{I}}_{i}^{end}$ respectively correspond to the lower and upper frequency bounds of the *i*-th detected signal, as shown in Equations (14)–(16). The binarization threshold γ is set to 0.8. The values in the predicted segmentation mask $\hat{y}$ are very close to one when that pixel contains a signal and close to zero otherwise. Therefore, unlike traditional threshold-based signal detection methods, the detection results are not sensitive to the choice of the binarization threshold.

(14) $${\hat{f}}_{i}^{lower}=f_{0}*{\hat{I}}_{i}^{start}$$

(15) $${\hat{f}}_{i}^{upper}=f_{0}*{\hat{I}}_{i}^{end}$$

(16) $$f_{0}=F_{s}/K_{fft}$$

where ${\hat{f}}_{i}^{lower}$ and ${\hat{f}}_{i}^{upper}$ represent the lower and upper frequency bounds of the *i*-th detected signal, respectively; $f_{0}$ corresponds to the frequency resolution selected. The ${\hat{f}}_{i}^{lower}$ and ${\hat{f}}_{i}^{upper}$ of the signal are equivalent to the estimation of carrier frequency and bandwidth, defined as

(17) $${\hat{f}}_{i}=\frac{1}{2}\left({\hat{f}}_{i}^{lower}+{\hat{f}}_{i}^{upper}\right)$$

(18) $${\hat{B}}_{i}={\hat{f}}_{i}^{upper}-{\hat{f}}_{i}^{lower}$$

## 4. Experiment

In this section, evaluation metrics and datasets applied in the subsequent experiments are presented. Then, several experiments are conducted to evaluate the performance of the proposed method.

### 4.1. Evaluation Metrics and Datasets

#### 4.1.1. Evaluation Metrics

**Figure 7:**
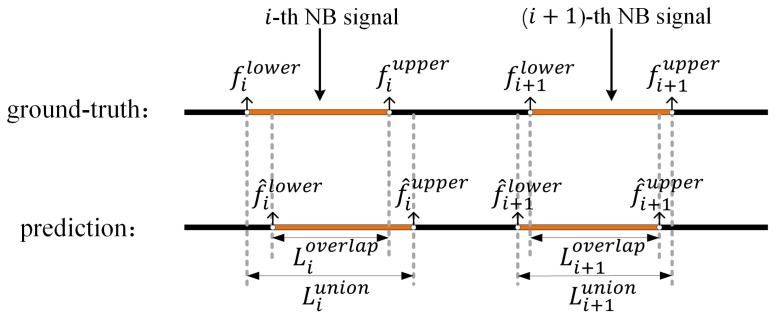
The length of overlap and union between the true signal spectrum position and estimated signal spectrum position.

The intersection-over-unit (IoU) is used to measure the correctness of individual signal detection result. The IoU measures the percentage of overlap between a predicted spectrum position and a true position in a dataset, which is defined as follow:

(19) $$IoU=\frac{L_{i}^{overlap}}{L_{i}^{union}}$$

where $L_{i}^{overlap}$ and $L_{i}^{union}$ describe the length of overlap and length of union between the true spectrum position and the estimated spectrum position of the *i*-th signal, as exhibited in Figure 7.

Typically, an IoU threshold η is applied to IoU to label a given prediction as true positive (*TP*) or false positive (*FP*). If the IoU between the true spectrum position and the estimated spectrum position (related to the carrier frequency and bandwidth of each signal) is greater than the IoU threshold η, the signal is considered to be detected. The IoU threshold η is set to 0.9 (unless otherwise specified). In this way, we can calculate the detection probability $P_{d}$ and false alarm probability $P_{f}$ to quantify the performance of the detection results. The $P_{d}$ and $P_{f}$ are computed by the following equations:

(20) $$P_{d}=\frac{N_{TP}}{N_{sig}}$$

(21) $$P_{f}=\frac{N_{FP}}{{\hat{N}}_{sig}}$$

where $N_{TP}$ denotes the number of signals which is correctly detected; $N_{FP}$ denotes the number of false alarm signals; $N_{sig}$ is the total number of signals in the true result; ${\hat{N}}_{sig}$ is the total number of signals in the detection result.

In addition, the mean absolute error $E_{avg}$ is also used to measure the estimation performance of carrier frequency $f_{c}^{i}$ and signal bandwidth $B^{i}$, which is defined as:

(22) $$E_{avg}=\frac{1}{2{\hat{N}}_{sig}}\sum_{i=1}^{{\hat{N}}_{sig}}\left(\frac{\left|f_{i}-{\hat{f}}_{i}\right|}{B_{i}}+\frac{\left|B_{i}-{\hat{B}}_{i}\right|}{B_{i}}\right)$$

where ${\hat{f}}_{i}$ and $f_{i}$ represent the predicted and true carrier frequency of the *i*-th signal, respectively; ${\hat{B}}_{i}$ and $B_{i}$ denote the predicted and true bandwidth of the *i*-th signal, respectively.

#### 4.1.2. Datasets

**Figure 8:**
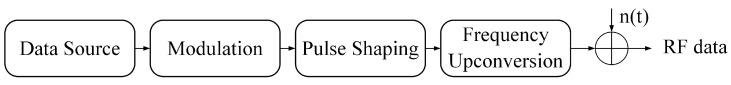
Single signal generation block.

Figure 8 shows a block diagram of our simulation framework used to generate a random single signal. Modulations used in simulation include 2ASK, BPSK, QPSK, 2FSK, and MSK. The root-raised cosine filter is used for pulse shaping (except 2FSK and MSK). The time duration range of each narrowband signal is [20 ms, 200 ms]; the carrier frequency range of each narrowband signal is [100 kHz, 3200 kHz]; the bandwidth range of each narrowband signal is [4 kHz, 110 kHz]. Each wideband RF capture consists of multiple narrowband signals, where the modulation type, duration, carrier frequency, and bandwidth of each narrowband signal are randomly selected from the ranges defined above. The number of narrowband signals contained in each wideband RF capture in the simulation is randomly chosen from [5,49]. Each generated RF capture is sampled at a sampling frequency of 6.4 MHz and a sampling duration of 200 ms. For different SNRs, the generated dataset is composed of 500 wideband RF captures, 80% of which are used for training and 20% for testing (validation).

To further demonstrate the effectiveness of our proposed method, a competition dataset provided by the “Smart Eye Cup” competition (https://www.landinn.cn/project/detail/1629978822137 (In Chinese), accessed on 10 February 2022) is also utilized. The goal of the competition is to achieve wideband signal detection in complex electromagnetic environments. The data samples are generated in a manner similar to the actual environment. Two signal styles are included in the dataset: constant signal and burst signal. The observation bandwidth is 3.2 MHz and the observation time is 200 ms or 2000 ms. Modulation types include BPSK, 2FSK, and GMSK. The SNR range is [4 dB, 25 dB].

### 4.2. Experimental Design and Baseline Methods

Three comprehensive experiments are conducted to verify the superiority of the proposed method for multi-signal detection. In the first experiment, the effect of parameter settings on the performance of the proposed method is investigated, including frequency resolution and the number of downsampling layers in the encoder network. In the second experiment, the performance of the proposed method is compared with existing methods. Furthermore, the validity of the Dice loss and PPM module is verified. To be fair, all methods perform the same pre-processing steps as described in Section 3 of the article. In the last experiment, a competition dataset is utilized to further demonstrate the effectiveness of the proposed method.

The training process is as follows: the network is trained for 100 epochs, the initial learning rate is 0.01 (dropped to 0.001 after 45 epochs for better learning convergence), and the mini-batch size is 20. During the learning process, the root mean square prop (RMSProp) algorithm is used to optimize the network. The proposed network is implemented by using the Pytorch framework and trained on a machine equipped with Nvidia Quadro RTX 4000 GPU and AMD R5-3600 CPU.

To demonstrate the effectiveness of the proposed method, the performance of the proposed SigdetNet is compared with two baselines: the LAD method in [26], and the FCN method in [36]. The parameter settings of these methods are based on the works in [26] and [36]. In [26], the localization of narrowband signals in the frequency domain is based on two thresholds. The lower and upper thresholds are set by two false alarm probabilities, respectively. The false alarm probability were $P_{lower,FA}=7\cdot 10^{-2}$ and $P_{upper,FA}=10^{-6}$. For the LAD method, no training is required, and the same testset as the proposed method is used for verification. For the FCN method, another deep learning-based method, the same dataset as the proposed method is used for training and testing.

## 5. Results and Discussion

### 5.1. Design Choices

In this section, the effects of various hyper-parameter choices on the performance of the proposed SigdetNet are discussed, including the number of FFT points $K_{fft}$ and the number of downsampling layers $N_{down}$.

**Figure 9:**
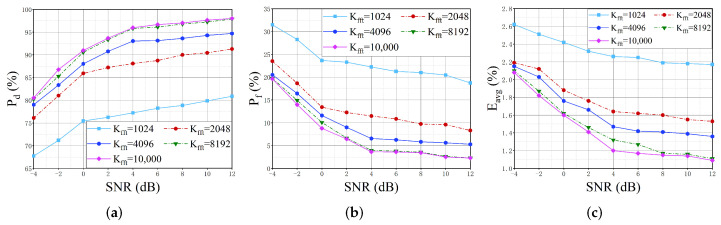
Performance variations with different numbers of FFT points. (**a**) detection probability; (**b**) false alarm probability; (**c**) mean absolute error of parameter estimation.

Number of FFT points $K_{fft}$: Figure 9 illustrates the variation of the detection probability $P_{d}$, false alarm probability $P_{f}$, and the mean absolute error $E_{avg}$ of parameter estimation under different FFT points $K_{fft}$, where $K_{fft}$ is set to 1024, 2048, 4096, 8192 and 10,000. As can be seen from Figure 9, the performance of the algorithm improves as the number of FFT points increases. However, when $K_{fft}$ is 1024, the performance declines sharply. Even with an SNR of 12dB, the $P_{d}$, $P_{f}$, and $E_{avg}$ are 80.89%, 18.73%, and 2.17% respectively, which are much lower than the performance with 10,000 FFT points. Firstly, fewer FFT points cause insufficient information provided by the input spectrum for network training, resulting in the network cannot capture more spectrum details. Secondly, the decrease of FFT points will lead to the reduction of frequency resolution $f_{0}$, while the estimation of signal bandwidth and carrier frequency is closely related to the frequency resolution, as shown in Equations (14)–(18), thus reducing the accuracy of parameter estimation. However, this does not mean that we need to increase the number of FFT points indefinitely in pursuit of optimal performance. It can be seen from Figure 9, the performance difference caused by $K_{fft}$ gradually decreases as the number of FFT points increases to a certain extent. Consequently, considering the trade-off between complexity and precision, the number of FFT points is set to 8192 in the following experiments.

**Figure 10:**
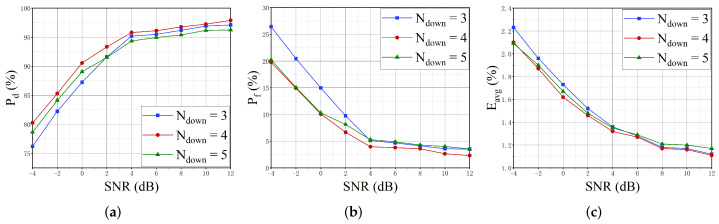
Performance variations with different numbers of downsampling layers. (**a**) detection probability; (**b**) false alarm probability; (**c**) mean absolute error of parameter estimation.

Number of downsampling layers $N_{down}$: Previous studies [56] have shown that the downsampling layers have an impact on the performance of segmentation tasks. Thus, an evaluation of the number of downsampling layers $N_{down}$ is performed. The max-pooling with 1×2 window and stride equal to 1 is used for downsampling the feature map by a factor of 2. Figure 10 illustrates the performance of the proposed SigdetNet with a different number of downsampling layers. $N_{down}$ is the number of downsampling layers, where $N_{down}$ is set to 3, 4, and 5, respectively, to reduce the size of the feature map to 1/8, 1/16, and 1/32 of the input. From Figure 10, it can be found that the SigdetNet performs best when the number of downsampling layers is 4. Although increasing the number of downsampling layers can improve the receptive field and reduce the network parameters, it also loses the origin information of the input power spectrum. Thus, four downsampling layers are utilized in our proposed SigdetNet to achieve the best performance in the following experiments.

### 5.2. Performance Comparison to Existing Methods

To demonstrate the superiority of the proposed method, comparisons with several representative signal detection methods, including the LAD method and the FCN method, are carried out. In addition, the impact of different loss functions on performance is investigated, including cross-entropy (CE) loss, Focal loss, and Dice loss. Furthermore, to verify the validity of PPM module, the performance of the network with PPM and without PPM is also compared.

**Figure 11:**
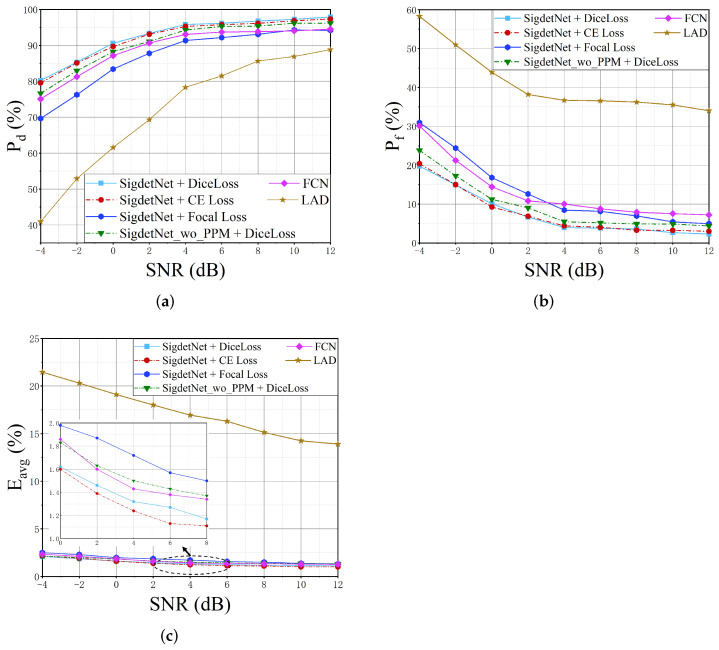
Performance comparison with existing methods. (**a**) detection probability comparison results; (**b**) false alarm probability comparison results; (**c**) comparison results of mean absolute error of parameter estimation.

Figure 11a–c, respectively shows the detection probability $P_{d}$, false alarm probability $P_{f}$ and the mean absolute error $E_{avg}$ of parameter estimation under different SNRs. In terms of the loss function, better performance can be obtained with the Dice loss, while Focal loss has the worst performance. For the reason that the Dice loss can deal with situations where there is an imbalance between the number of signal and background pixels.

Compared with the LAD method, the proposed SigdetNet and the FCN method are significantly superior. The superior performance of the deep learning-based method may be attributed to the sophisticated feature extraction procedure and the superior learning ability of the deep neural networks. With the increase of SNR, the detection probability of the LAD method can reach more than 80%, but its false alarm probability is also higher. The LAD method is a threshold-based detection method, which is difficult to set thresholds due to the ubiquitous noise and fluctuation. When the threshold is set lower, the detection probability increases, but the false alarm probability also increases. Furthermore, the LAD method is difficult to accurately detect the frequency boundaries of signals due to noise fluctuation, so it also performs poorly in parameter estimation.

Compared with the FCN method which also adopts deep learning, the proposed method achieves better performance. The proposed SigdetNet can reach over 90% detection probability when the SNR is larger than 0 dB, and can achieve over 95% detection probability when the SNR is at 4 dB. However, the best detection probability of FCN is 94.5% when the SNR is at 12 dB. When SNR is higher than −4 dB, the false alarm probability of the proposed method can maintain below 20%, while the false alarm probability of FCN is 30% when SNR is −4 dB. The main reasons for the better performance of the proposed method include: firstly, the SigdetNet uses ResNet as the backbone network, and residual learning can solve the problem of vanishing gradients to train a deeper network; secondly, the use of PPM module can fuse multi-scale feature maps to effectively extract features with context information. On the contrary, the FCN method lack of ability to infer from the context, which may cause false detections or unclear boundary segmentation due to noise fluctuations. Context information is beneficial for signal detection, for example, the power spectrum of a 2FSK signal with a large modulation index has in-band splitting. The energy between the two spectral peaks of 2FSK is low, and if out of context information, the pixels between the two spectral peaks may be misjudged as background, resulting in one signal being falsely detected as multiple signals.

To further demonstrate the validity of the proposed method, the network is trained without PPM and obtains the SigdetNet_wo_PPM curve in Figure 11. The results show that the performance of the network with the PPM module is better than that without the PPM module. When the SNR is below 0 dB, SigdetNet with a PPM module can achieve a 2∼3% improvement in detection probability and false alarm probability compared to that without PPM. Table 1 shows the complexity comparison of the SigdetNet with the PPM module and without the PPM module in terms of floating-point operations (FLOPs) and network parameters. It can be seen that the added PPM module does not significantly increase the complexity.

### 5.3. Performance on the Competition Dataset

**Figure 12:**
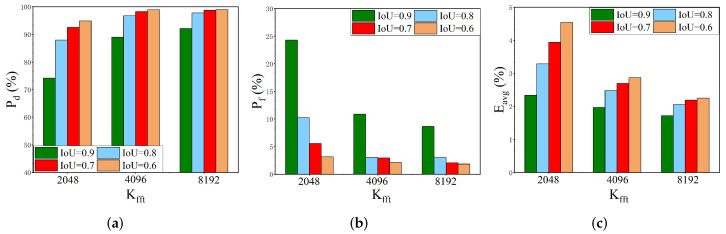
Performance results on the competition dataset. (**a**) detection probability; (**b**) false alarm probability; (**c**) mean absolute error of parameter estimation.

**Figure 13:**
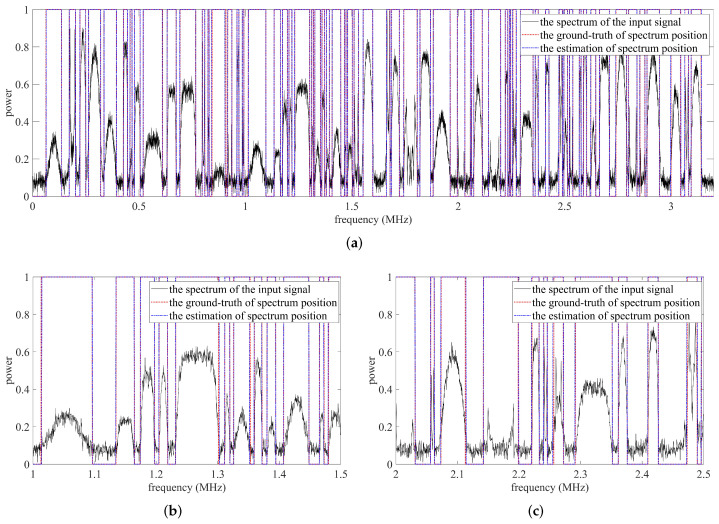
Predicted results for the first example. (**a**) The whole band of the first prediction example. (**b**) The sub-band_1 of the first prediction example. (**c**) The sub-band_2 of the first prediction example.

**Figure 14:**
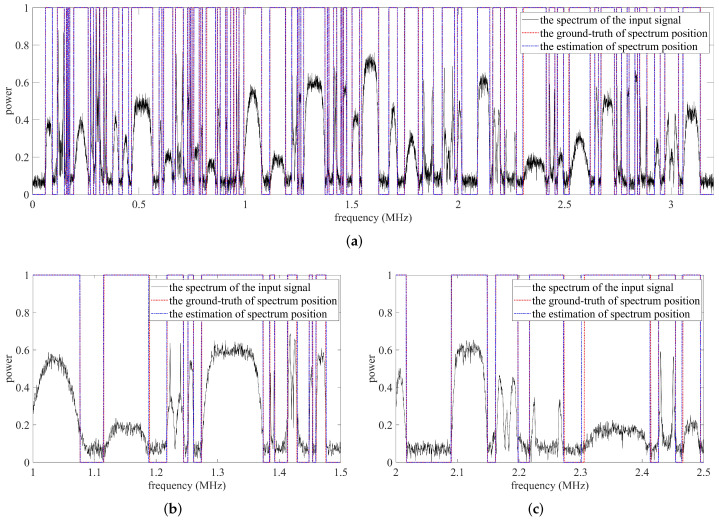
Predicted results for the second example. (**a**) The whole band of the second prediction example. (**b**) The sub-band_1 of the second prediction example. (**c**) The sub-band_2 of the second prediction example.

In order to further verify the effectiveness and applicability of the proposed method, a competition dataset is utilized. The dataset includes 500 wideband RF captures, each RF capture containing multiple narrowband signals, where each narrowband signal has a different modulation type and signal-to-noise ratio. The narrowband signals in a wideband RF capture include constant and burst signals. In previous experiments, the performance of the proposed method was evaluated when the IoU threshold was fixed at 0.9. In this section, the performance under different IoU threshold η is analyzed, where $\eta \in \left[0.6,0.7,0.8,0.9\right]$, as shown in Figure 12. The proposed SigdetNet maintains a satisfactory detection performance, which is similar to the previous results. Naturally, when the IoU threshold increases, the detection probability and false alarm probability deteriorate because most predictions are discarded. Two prediction examples are shown in Figure 13 and Figure 14 respectively, and there are detailed results for the two subbands below each prediction example. The results show the proposed method achieves good effectiveness in different types of power spectrums, almost all signals in the spectrum can be detected, and their lower and upper frequency positions can be obtained at the same time. In practice, the number and bandwidth of signals in each wideband RF capture are time-varying, with some signal bandwidths spanning a wide range, while others are very narrow. In Figure 13 and Figure 14, the results show that the proposed method can also deal with these problems well. Although the detection results demonstrate the practicability of the proposed method, there are still some problems. For example, as shown in Figure 14c, spectral boundaries cannot be exactly estimated for weak signals.

## 6. Conclusions

In this paper, the deep learning technology is applied to solve the non-cooperative multi-signal detection problem, that is, to jointly detect the presence of signals as well as estimate their center frequencies and bandwidths. The proposed framework, named SigdetNet, includes signal pre-processing, signal enhancement, feature extraction using NN, and post-processing. In the signal pre-processing stage, Welch’s method is utilized to reduce the variance of the PSD estimation. Then, a logarithmic transformation is also applied for signal enhancement. In particular, a convolutional encoder-decoder network with the embedding pyramid pooling module is constructed to extract informative features related to signal detection from multi-scale. Extensive simulation results demonstrated that our proposed method is superior to other benchmark schemes, e.g., the LAD method and the FCN method. Interesting avenues for the future include (i) conducting detailed studies to determine the most suited network structure for signal detection applications, and (ii) building custom-made denoisers to improve performance under low SNR.

## Figures and Tables

**Table 1:** The comparison of complexity.

Model	FLOPs	Parameters
SigdetNet	335.2928 M	0.3624 M
SigdetNet without PPM	334.1107 M	0.3618 M

## Data Availability

Not applicable.

## Abbreviations

The following abbreviations are used in this manuscript:

CR	Cognitive radio
PSD	Power spectral density
IoT	Internet of Things
ED	Energy detection
LAD	Double-thresholding
FCME	Forward consecutive mean excision
SNR	Signal to noise ratio
NN	Neural network
NLP	Natural language processing
CNN	Convolutional neural network
GRU	Gate recurrent unit
SSD	Single shot multibox detector
Faster-RCNN	Faster region-based convolutional neural network
RFI	RF interference
FCN	Full convolutional network
PSPNet	Pyramid scene parsing network
PSD	Power spectral density
PPM	Pyramid pooling module
ASK	Amplitude shift keying
PSK	Phase shift keying
FSK	Frequency shift keying
GMSK	Gaussian minimum shift keying
RF	Radio frequency
AWGN	Additive White Gaussian Noise
FFT	Fast Fourier transform
BN	Batch normalized
ReLU	Rectified linear unit
CE	Cross-entropy
IoU	Intersection-over-unit
TP	True positive
FP	False positive
RMSProp	Root mean square prop
FLOPs	Floating-point operations
